# Associations between blood cadmium and endocrine features related to PCOS-phenotypes in healthy women of reproductive age: a prospective cohort study

**DOI:** 10.1186/s12940-021-00749-4

**Published:** 2021-05-22

**Authors:** Keewan Kim, Anna Z. Pollack, Carrie J. Nobles, Lindsey A. Sjaarda, Jessica R. Zolton, Jeannie G. Radoc, Enrique F. Schisterman, Sunni L. Mumford

**Affiliations:** 1 Epidemiology Branch, Division of Intramural Population Health Research, Eunice Kennedy Shriver National Institute of Child Health and Human Development, 6710B Rockledge Drive MSC 7004, Maryland 20892 Bethesda, USA; 2 George Mason University, 22030 Fairfax, Virginia USA; 3 Program in Reproductive Endocrinology and Gynecology, Eunice Kennedy Shriver National Institute of Child Health and Human Development, 20892 Bethesda, Maryland USA

**Keywords:** Cadmium, Testosterone, Anti-Müllerian hormone (AMH), Sex hormone-binding globulin (SHBG), Polycystic ovary syndrome (PCOS)

## Abstract

**Background:**

Cadmium is an endocrine disrupting chemical that affects the hypothalamic-pituitary-gonadal axis. Though evidence suggests its potential role in altering androgen synthesis and metabolic pathways that are characteristic of polycystic ovary syndrome (PCOS), its relation in healthy women of reproductive age is largely unknown. As women with mild sub-clinical features of PCOS who do not meet the diagnostic criteria of PCOS may still experience reduced fecundability, investigating associations between cadmium and PCOS-phenotypes among healthy women may provide unique insight into the reproductive implications for many on the PCOS spectrum. Therefore, the objective of this study was to evaluate associations between cadmium and androgens, anti-Müllerian hormone (AMH), and metabolic markers in women of reproductive age.

**Methods:**

This was a prospective cohort study of 251 healthy premenopausal women without self-reported PCOS (mean age 27.3 years and BMI 24.1 kg/m^2^). Cadmium was measured in blood collected at baseline. Reproductive hormones and metabolic markers were measured in fasting serum 8 times per menstrual cycle for 2 cycles. Linear mixed models and Poisson regression with a robust error variance were used to examine associations between cadmium and reproductive hormones and metabolic markers and anovulation, respectively.

**Results:**

Median (interquartile range) blood cadmium concentrations at baseline were 0.30 (0.19–0.43) µg/L. Higher levels of testosterone (2.2 %, 95 % confidence interval [CI] 0.4, 4.1), sex hormone-binding globulin (2.9 %, 95 % CI 0.5, 5.5), and AMH (7.7 %, 95 % CI 1.1, 14.9) were observed per 0.1 µg/L increase in cadmium concentrations. An 18 % higher probability of a mild PCOS-phenotype (95 % CI 1.06, 1.31), defined by a menstrual cycle being in the highest quartile of cycle-averaged testosterone and AMH levels, was also found per 0.1 µg/L increase in cadmium levels. No associations were observed for insulin and glucose. These findings were consistent even after analyses were restricted to non-smokers or further adjusted for dietary factors to account for potential sources of exposure.

**Conclusions:**

Overall, among healthy reproductive-aged women, cadmium was associated with endocrine features central to PCOS, but not with metabolic markers. These suggest its potential role in the hormonal milieu associated with PCOS even at low levels of exposure.

**Supplementary Information:**

The online version contains supplementary material available at 10.1186/s12940-021-00749-4.

## Background

Cadmium is a non-essential toxic metal that is ubiquitously found in the environment and does not have a beneficial role in human health. Inhalation of cigarette smoke is one of the major sources of exposure to cadmium, whereas consumption of contaminated foods, such as shellfish, rice, grains, and vegetables, is the main source of exposure among non-smokers [1]. It is an endocrine disrupting chemical that exerts estrogenic effects and has been shown to affect the hypothalamic-pituitary-gonadal (HPG) axis [2], which could result in reproductive dysfunction [3]. Toxicity of cadmium that influences reproductive hormones was also evident in our prior study among healthy women of reproductive age with low levels of cadmium exposure. Specifically, blood cadmium concentrations were associated with higher levels of estradiol [4] and lower levels of follicle-stimulating hormone (FSH) though did not affect anovulation [5], suggesting that cadmium may affect tightly regulated reproductive hormone levels even at low levels of exposure.

Cadmium was previously hypothesized to have a role in influencing androgens and metabolic features. In experimental studies, exposure to high levels of cadmium has been shown to influence gonadotropic hormone secretion [6, 7], induce alterations in androgen biosynthesis [8], and block binding of androgens to the receptor in human prostate cancer cells [9]. Cadmium has been further shown to induce apoptosis in pancreatic beta-cells in mice [10] and hyperglycemia in rats [11], suggesting its potential effect on metabolism.

Though evidence supports an influence of cadmium on androgens and metabolic features, factors commonly associated with polycystic ovary syndrome (PCOS), less is known about the impacts of exposures among healthy reproductive-aged women. Links between PCOS-phenotypes in otherwise healthy women and manifestation of overt PCOS are not always clear. However, women with a mild PCOS-phenotype who do not meet the diagnostic criteria of PCOS have still been shown to experience reduced fecundability [12]. As such, investigating associations between cadmium exposure and PCOS-phenotypes among healthy women may provide unique insight into the reproductive implications for many on the PCOS spectrum. Moreover, given that smoking is one of the primary sources of exposure to cadmium associated with significant reproductive harm, it is critical to assess the role of cadmium among non-smoking populations. Therefore, the objective of this study was to evaluate associations between blood cadmium concentrations and androgens, anti-Müllerian hormone (AMH), and metabolic markers in healthy women of reproductive age with low levels of cadmium exposure, primarily non-smokers.

## Materials and methods

### Study design and sample collection

This was a prospective cohort study of healthy regularly menstruating women enrolled in the BioCycle Study which was conducted in western New York in 2005–2007. The study design was previously described in detail [13]. In brief, women 18–44 years of age with self-reported menstrual cycle lengths of 21–35 days for at least 6 months prior to recruitment and self-reported body mass index (BMI) of > 18 or < 35 kg/m^2^ at screening were eligible for the study. Women who used hormonal contraceptives in the past 3 months prior to screening, were pregnant or breastfeeding in the past 6 months, had a history of ovulatory disorders (e.g., diagnosis of PCOS) or endocrine dysfunction were excluded.

Fasting blood specimens were obtained from each participant up to 8 times per cycle during participant’s visits at specific phases of the menstrual cycle, including the second day of menstruation (1 visit), the mid follicular phase (1 visit), the periovulatory phase (3 visits), and during the early, mid-, and late luteal phase (3 visits). Fertility monitors (Clearblue Easy Fertility Monitor; Inverness Medical, Waltham, MA) and personal cycle length histories were used to determine the timing of visits [14]. Overall, data for 2 cycles from 250 women and 1 cycle from 9 women were collected, with 94 % of the participants completing at least 7 clinic visits per cycle. The University at Buffalo Health Sciences Institutional Review Board (IRB) served as the IRB designated by the National Institutes of Health under a reliance agreement and approved the study. All study participants provided written informed consent.

### Blood cadmium analysis

At the screening visit, whole blood was collected in purple-top Vacutainer tubes (Becton, Dickinson and Company, Franklin Lakes, NJ) that contained ethylendiaminetetraacetic acid (EDTA). Cadmium concentrations were determined in 252 out of 259 women enrolled in the study using inductively coupled plasma mass spectrometry at the Division of Laboratory Sciences of the National Center for Environmental Health at the Centers for Disease Control and Prevention (Atlanta, GA). Approximately 25 % of the samples were below the limit of detection (0.20 µg/dL) and machine read values were not substituted to minimize potential bias [15]. The interassay coefficient of variation (CV) was 4.3 % at 2.04 µg/dL. One individual had cadmium concentrations greater than 3 standard deviations (SDs) from the mean of the overall sample and was therefore excluded from this analysis; the final sample included 251 women.

### Measurement of reproductive hormones and metabolic markers

Total testosterone (ng/dL) was measured in serum samples by a liquid chromatography/tandem mass spectrometry using a Shimadzu Prominence Liquid Chromatograph (Shimadzu Scientific Instruments, Inc., Columbia, MD) with an ABSceix 5500 tandem mass spectrometer (AB SCIEX, Framingham, MA) at the University of Minnesota, Minneapolis, MN. Increased sensitivity was obtained using Mobile Phase B (100 % acetonitrile) with a low standard of 4 ng/dL added to the standard curve. Serum AMH (ng/mL) was measured using the GEN II ELISA assay (Beckman Coulter, Inc, Brea, CA). Concentrations of sex hormone-binding globulin (SHBG, nmol/L) were determined using solid-phase competitive chemiluminescent enzymatic immunoassays (Specialty Laboratories Inc., Valencia, CA) on a DPC Immulite 2000 analyzer (Siemens Medical Solutions Diagnostics, Deerfield, IL) at the Kaleida Health Center for Laboratory Medicine, Buffalo, NY [13]. The CVs were < 7 % for total testosterone, < 12 % for AMH, and < 10 % for SHBG. Free testosterone was calculated as 24.00314 × (total testosterone/log_10_SHBG) – 0.0499 × (total testosterone)^2^ [16] and free androgen index as 100 × (total testosterone/SHBG), based on standardized methods described previously [17].

Metabolic markers were determined in serum and plasma samples. Insulin (µU/mL) was measured in serum using a competitive chemiluminescent enzymatic immunoassay and glucose (mg/dL) was determined in plasma using a hexokinase-based method on a Beckman LX20 autoanalyzer (Beckman Coulter, Inc, Brea, CA). The CVs were < 8 % for insulin and < 3 % for glucose. Homeostatic Model Assessment of Insulin Resistance (HOMA-IR), a marker of insulin resistance, was calculated as (fasting insulin × fasting glucose)/22.5, and described elsewhere [18].

### Covariate assessment

At baseline, participants completed questionnaires on demographic characteristics (e.g., race, education), lifestyle factors (e.g., cigarette smoking, physical activity, diet), and reproductive history (e.g., past oral contraceptive use, parity). Physical activity was assessed using the International Physical Activity Questionnaire (IPAQ) long form 2002 [19], and the level of physical activity was estimated as high, moderate, and low based on standard IPAQ cutoffs. Dietary factors were assessed using a food frequency questionnaire which captured food intakes during the previous 6 months (Fred Hutchinson Cancer Center, Seattle, WA). Weight and height of participants were measured by trained staff according to standardized protocols to calculate BMI (kg/m^2^).

### Statistical analysis

Demographic and lifestyle factors (mean ± SD or n [%]) were compared by tertile of blood cadmium levels, using Fisher’s exact tests and ANOVAs. Cadmium concentrations were rescaled for regression analyses given low levels of exposure in participants. Associations between blood cadmium concentrations (per 0.1 µg/L increase) and log-transformed reproductive hormones and metabolic markers were evaluated using linear mixed models, accounting for repeated measures within woman during the cycle and across cycles. Models were adjusted for age (years), BMI (kg/m^2^), race (white, black, or other), and parity (nulliparous vs. parous) (Model 1). Results were compared with models that additionally adjusted for smoking status (current vs. no or former smokers) (Model 2). In a separate analysis, models were further adjusted for consumption of either rice, grains, and leafy vegetables in order to investigate the potential impact of dietary factors that might be related to cadmium exposure and PCOS [1, 20]. Results were presented as percent differences and 95 % confidence intervals (CIs). All analyses were repeated only among non-smokers.

Associations between blood cadmium concentrations and a mild PCOS-phenotype were further examined by considering both total testosterone and AMH. Specifically, using cycle-averaged levels of total testosterone and AMH, cycles were categorized into a mild PCOS-phenotype (i.e., both testosterone and AMH being in the upper 75th percentile) versus no (i.e., either or both testosterone and AMH in the lower 75th percentile). Poisson regression with a robust error variance was used to estimate risk ratios (RRs) and 95 % CIs. Models were adjusted for the same covariates listed above. SAS version 9.4 (SAS Institute, Cary, NC) was used for all statistical analyses.

## Results

Median (interquartile range; IQR) levels of blood cadmium were 0.30 (0.19–0.43) µg/L in 251 participants (Table 1). Women in the highest tertile of blood cadmium concentrations tended to be older (mean ± SD; highest tertile 29.9 ± 8.2 years of age, lowest tertile 25.7 ± 7.8) and self-identified as black or other than white or black for race/ethnicity. 4 % of women were current smokers and smoking status was positively correlated with blood cadmium concentrations (*P* = 0.01). No other demographic or lifestyle characteristics were correlated with blood cadmium levels.

**Table 1:** Demographic and lifestyle characteristics by blood cadmium concentrations in 251 participants in the analysis

Mean ± SD or n (%)	Overall	Tertile 1	Tertile 2	Tertile 3	*P*-value
N	251	82	88	81	
Blood cadmium (µg/L; median [IQR])	0.30 (0.19–0.43)	0.15 (0.12–0.19)	0.30 (0.26–0.33)	0.50 (0.44–0.68)	
Demographic factors					
Age, year	27.3 ± 8.2	25.7 ± 7.8	26.4 ± 7.5	29.9 ± 8.2	0.002
BMI, kg/m^2^	24.1 ± 3.8	24.1 ± 3.8	24.1 ± 3.9	24.0 ± 3.8	0.984
Race					0.001
White	149 (59)	61 (74)	50 (57)	38 (47)	
Black	51 (20)	15 (18)	19 (22)	17 (21)	
Other	51 (20)	6 (7)	19 (22)	26 (32)	
High school education or less	31 (12)	9 (11)	11 (13)	11 (14)	0.862
Lifestyle factors					
Physical activity					0.088
High	139 (55)	11 (13)	5 (6)	8 (10)	
Moderate	88 (35)	21 (26)	32 (36)	35 (43)	
Low	24 (10)	50 (61)	51 (58)	38 (47)	
Current smoking	10 (4)	0 (0)	3 (3)	7 (9)	0.011
Parous	65 (26)	18 (23)	23 (27)	24 (30)	0.823
Oral contraceptive use	136 (55)	44 (54)	50 (57)	42 (53)	0.959

*BMI* body mass index, *IQR *interquartile range, *SD *standard deviation

Positive associations between blood cadmium concentrations and reproductive hormones that are characteristic of PCOS were observed (Table 2). Specifically, a 0.1 µg/L increase in blood cadmium concentrations was associated with 2.2 % higher total testosterone (95 % CI 0.4, 4.1), 2.9 % higher SHBG (95 % CI 0.5, 5.5), and 7.7 % higher AMH (95 % CI 1.1, 14.9), adjusting for age, BMI, race, parity, and cigarette smoking (Model 2). Unlike hormones, no associations were indicated for blood cadmium with metabolic markers, including insulin, glucose, and HOMA-IR. No appreciable changes were found after further adjusting for dietary factors that might be related to cadmium exposure and PCOS, including consumption of rice, grains, and green vegetables (Table 3). Our results were also consistent after analyses were restricted to non-smokers (Supplementary Table 1).

**Table 2:** Associations between blood cadmium concentrations (per 0.1 µg/L) and reproductive hormones and metabolic markers

	Unadjusted	Model 1	Model 2
	%Difference (95 % CI)	%Difference (95 % CI)	%Difference (95 % CI)
Reproductive hormones			
Testosterone (ng/dL)	0.5 (-1.3, 2.3)	2.0 (0.3, 3.7)^*^	2.2 (0.4, 4.1)^*^
Free testosterone (ng/dL)	-0.4 (-2.2, 1.3)	1.3 (-0.3, 2.8)	1.3 (-0.3, 3.0)
Free androgen index	-2.6 (-5.0, -0.1)^*^	-0.3 (-2.5, 2.0)	-0.8 (-3.1, 1.6)
SHBG (nmol/L)	3.2 (0.8, 5.7)^**^	2.2 (-0.1, 4.5)	2.9 (0.5, 5.5)^*^
AMH (ng/mL)	-2.0 (-8.5, 5.0)	5.9 (-0.3, 12.4)	7.7 (1.1, 14.9)^*^
Metabolic markers			
Insulin (µU/mL)	-2.1 (-4.0, -0.1)^*^	-1.6 (-3.4, 0.2)	-1.3 (-3.2, 0.6)
Glucose (mg/dL)	0.1 (-0.2, 0.4)	0.0 (-0.3, 0.3)	0.1 (-0.3, 0.4)
HOMA-IR	-1.9 (-4.0, 0.1)	-1.6 (-3.5, 0.3)	-1.2 (-3.3, 0.8)

Model 1: Adjusted for age, BMI, race, and parity

Model 2: Adjusted for age, BMI, race, parity, and smoking

^*^P < 0.05, ^**^P < 0.01

*AMH *anti Müllerian hormone, *BMI *body mass index, *CI *confidence interval, *HOMA-IR *homeostatic model assessment of insulin resistance, *SHBG *sex hormone-binding globulin

**Table 3:** Associations between blood cadmium concentrations (per 0.1 µg/L) and reproductive hormones and metabolic markers, adjusting for dietary factors

	Adjusted for rice	Adjusted for grains	Adjusted for green vegetables
	%Difference (95 % CI)	%Difference (95 % CI)	%Difference (95 % CI)
Reproductive hormones			
Testosterone	2.2 (0.4, 4.1)^*^	2.2 (0.4, 4.1)^*^	2.2 (0.4, 4.1)^*^
Free testosterone	1.3 (-0.4, 3.0)	1.3 (-0.4, 3.0)	1.3 (-0.4, 3.0)
Free androgen index	-0.7 (-3.0, 1.7)	-0.8 (-3.2, 1.6)	-0.8 (-3.1, 1.6)
SHBG	2.9 (0.4, 5.4)^*^	3.0 (0.6, 5.6)^*^	3.0 (0.5, 5.5)^*^
AMH	6.1 (-0.5, 13.1)	7.6 (0.9, 14.8)^*^	7.6 (0.9, 14.7)^*^
Metabolic markers			
Insulin	-1.3 (-3.2, 0.7)	-1.3 (-3.2, 0.6)	-1.3 (-3.2, 0.6)
Glucose	0.1 (-0.2, 0.4)	0.1 (-0.3, 0.4)	0.1 (-0.2, 0.4)
HOMA-IR	-1.2 (-3.2, 0.9)	-1.2 (-3.2, 0.8)	-1.2 (-3.2, 0.8)

Note: All models were adjusted for age, BMI, race, parity, and smoking. ^*^P<0.05

*AMH* anti Müllerian hormone, *BMI* body mass index, *CI* confidence interval, *HOMA-IR* homeostatic model assessment of insulin resistance, *SHBG* sex hormone-binding globulin

Among 480 cycles from women who had both total testosterone and AMH measurements, 56 (11.7 %) were classified as a mild PCOS-phenotype, defined by a cycle being in the highest quartile of total testosterone and AMH (Table 4). Of remaining cycles, 64 cycles were in the lower 75th percentile of testosterone, 56 cycles were in the lower 75th percentile of AMH, and 304 cycles were in the lower 75th percentile for both testosterone and AMH. Our data suggested that each 0.1 µg/L increase in blood cadmium levels was associated with an 18 % higher probability of a mild PCOS-phenotype (RR 1.18; 95 % CI 1.06, 1.31; Model 2).

**Table 4:** Associations between blood cadmium concentrations and PCOS-phenotype defined by cycles being in the highest quartile of total testosterone and AMH

	RR (95 % CI)
Unadjusted	1.05 (0.92, 1.20)
Model 1	1.16 (1.04, 1.29)^**^
Model 2	1.18 (1.06, 1.31)^**^

Model 1: Adjusted for age, BMI, race, and parity

Model 2: Adjusted for age, BMI, race, parity, and smoking

^**^P < 0.01

*AMH *anti Müllerian hormone, *BMI *body mass index, *CI *confidence interval, *PCOS *polycystic ovary syndrome, *RR *risk ratio

## Discussion

Among healthy premenopausal women without a diagnosis of PCOS, higher blood cadmium concentrations were associated with elevated testosterone and AMH levels, key endocrine markers that are central to PCOS. Our data also supported that increasing blood cadmium concentrations were associated with higher concentrations of SHBG. In contrast, no associations were observed between cadmium and metabolic markers, including insulin and glucose. Our findings were consistent among non-smokers or even after adjusting for certain food intakes, including rice, grains, and green vegetables, that are potentially important in the association between cadmium exposure and PCOS-related features. Overall, these findings suggest a potential role of cadmium in the hormonal milieu, but not metabolic pathway, associated with PCOS among healthy women of reproductive age, even at low levels of exposure.

Our findings of increased testosterone levels with higher blood cadmium concentrations are in line with prior studies among postmenopausal women, where high levels of blood [21] and urinary cadmium [22] were associated with elevated serum testosterone concentrations. However, our findings are in contrast to more recent studies, which observed lower concentrations of testosterone with higher levels of blood cadmium (mean 1.90 µg/L) in Chinese men [23] and with increasing urinary cadmium in pre- (mean 2.33 µg/L) and postmenopausal (mean 3.21 µg/L) Japanese women [24], though levels of cadmium measured in these studies were substantially higher than in our data. Cadmium may exert reproductive toxicity via influences on the HPG-axis, particularly through effects on the pituitary gland, altering gonadotropin pulsatility and thereby influencing the release of gonadotropic hormones, and subsequently altering testosterone levels [3]. Indeed, we previously observed a decrease in FSH with increasing blood cadmium levels in this cohort [5], further supporting this mechanism. Alternatively, our findings could be in part explained by the effect of cadmium on the androgen receptors at the ovary. Androgen-like activity of cadmium has been suggested in experimental studies where cadmium was shown to trigger an androgen-specific reporter gene in human prostate epithelial cells in the presence of the androgen receptor [25] and block the binding of androgens to the receptor in human prostate cancer cells [9]. Thus, it is plausible that cadmium may share biological activity with androgens, bind to androgen receptors at ovary, and stimulate testosterone production as observed in our data.

A positive association between cadmium and SHBG was indicated in our data and this was expected as increases in sex steroids are linked to increases in SHBG. Alternatively, the observed association may be related to cadmium’s ability to bind to SHBG. Specifically, cations (e.g., Ca^2+^, Zn^2+^) including cadmium are able to bind to SHBG, competing with estradiol and androgens [26], and this in turn can stimulate SHBG production in the liver [27]. This potential mechanism is in line with our observation for testosterone as well, since binding of cadmium to SHBG may interfere with binding of testosterone to SHBG which subsequently can lead to small increases in testosterone levels in circulation. Increased SHBG levels were also observed in men at slightly higher levels of blood cadmium (median 0.38 µg/L, 10th -90th percentile 0.05–1.27 [28] or weighted median 0.40 µg/L, IQR 0.20–0.70 [29]) than in our cohort (median 0.30, IQR 0.19–0.43). Though cadmium levels measured in the general population [28, 29] as well as in our cohort are much lower than in the prior experimental studies [26], taken together this evidence may point to the potential contribution of cadmium on influencing SHBG.

AMH is produced in the granulosa cells of the small preantral and antral follicles and is relatively higher among women with PCOS [30]. Our finding is consistent with the result from a cross-sectional study among pregnant women, where a positive relationship was suggested for blood cadmium and AMH levels [31]. However, our result differs from a recent study among premenopausal women, which reported an inverse association between blood cadmium and AMH concentrations, though mean age of participants in that study were higher than ours (mean ± SD, 36.2 ± 3.9 vs. 27.3 ± 8.2) [32]. Specific mechanisms are unclear; however, our observation of an increase in AMH with higher cadmium concentrations could be related to the effect through damage of granulosa cells [33] or alterations in the blood-follicle-barrier due to cadmium toxicity, as disruption of the follicle membrane integrity may potentially lead to an increase in the release of AMH from the follicles [34, 35].

High levels of cadmium have induced hyperglycemia [11] and apoptosis in pancreatic beta-cells in rats and mice [10], suggesting a potential role in metabolic dysregulation. However, despite the well-established toxicological effects of cadmium on metabolic function in animal studies, epidemiological evidence is inconsistent perhaps due to differences in population, levels of exposure, and lifestyle factors that affect the body burden of cadmium. In a cross-sectional study using a large sample from the National Health And Nutrition Examination Survey (NHANES) 1988–1994 (≥ 40 years old), urinary cadmium (mean 0.92 and 0.63 µg/g creatinine among smokers and non-smokers, respectively) was associated with impaired fasting glucose and elevated risk of diabetes [36]. On the other hand, a study among older women (≥ 64 years old) with comparable cadmium concentrations to ours (median 0.34 µg/L for blood cadmium and 0.36 µg/g creatinine for urine cadmium) found no associations with insulin sensitivity and glucose tolerance [37]. A more recent study using data from the NHANES 2001–2014 also reported no associations between urinary cadmium and risk of metabolic syndrome in women (≥ 20 years) [38], consist with our null findings. Though metabolic dysregulation is a frequently observed feature among women with PCOS [39, 40], our data suggest a minimal effect of low levels of cadmium exposure in relation with changes in metabolic markers in healthy women.

In cross-sectional studies which compared serum cadmium concentrations between women with and without PCOS, no differences in cadmium levels were observed [41, 42]. In our study where approximately 12 % of the total cycles were classified as a mild PCOS-phenotype using testosterone and AMH measurements, our data showed that higher cadmium concentrations were associated with a higher probability of a mild PCOS-phenotype. It is important to note that even though our findings suggest that cadmium exposure may contribute to alterations in key hormonal characteristics of PCOS, it does not imply direct associations with PCOS as our women have never been diagnosed with PCOS and other factors may play a more critical role in leading to manifestation of overt PCOS.

Several strengths and limitations in our study are noteworthy. Cadmium was determined in a single blood sample collected at baseline. Unlike urinary cadmium which reflects long-term exposure, blood cadmium is a marker of a short-term exposure and thus there is a possibility that an already altered hormonal milieu or metabolic function could have affected cadmium metabolism. However, multiple prospective measurements of serum reproductive hormones and metabolic markers across the menstrual cycle may make this case less likely. Blood cadmium measured in our study was lower than in other studies [36, 37] possibly due to a small proportion of current smokers in our data (4 %) which further enables us to distinguish between the deleterious effects of smoking and cadmium exposure. Nevertheless, blood cadmium levels in our study were comparable to that measured among women in NHANES 2005–2006 (geometric mean 0.33, 95 % CI 0.31, 0.35) [43] or non-smoking women aged 20–44 years in NHANES 2003–2006 (geometric mean 0.34, 95 % CI 0.32, 0.36) [44]. Importantly, the potential impact of dietary factors that might be related to cadmium exposure and PCOS [1, 20] have been accounted during the analysis and consistent results were observed, supporting robustness of our findings between cadmium exposure and PCOS-related endocrine and metabolic features.

## Conclusions

Overall, our findings suggest a potential association between increasing levels of cadmium with higher testosterone, AMH, and SHBG concentrations among healthy women of reproductive age, largely non-smokers with low levels of cadmium exposure. Given the known link between these hormonal factors and PCOS, the most common endocrine disorder in women [45], our findings are important in understanding potential factors that affect the continuum of PCOS. As women with mild sub-clinical features of PCOS may suffer from ovulatory insufficiency and other factors related to impaired fecundability [12], our findings also highlight the need to mitigate exposure to this ubiquitous environmental contaminant through modifiable lifestyle changes for women’s reproductive health.

## Supplementary Information

**Additional file 1.**

## Data Availability

The datasets used and/or analyzed during the current study are available from the corresponding author on reasonable request. Some of baseline demographic data are publicly available from the NICHD’s database (Data And Specimen Hub or DASH) at https://dash.nichd.nih.gov.

## Abbreviations

AMH: Anti-Müllerian hormone
BMI: Body mass index
CI: Confidence interval
CV: Coefficient of variation
FSH: Follicle-stimulating hormone
HPG axis: Hypothalamic-pituitary-gonadal axis
IPAQ: International Physical Activity Questionnaire
LH: Luteinizing hormone
NHANES: National Health And Nutrition Examination Survey
SHBG: Sex hormone-binding protein
PCOS: Polycystic ovary syndrome
RR: Risk ratio
