# Supplementary material for: Associations between blood cadmium and endocrine features related to PCOS-phenotypes in healthy women of reproductive age: a prospective cohort study
Source: Environ Health. 2021 May 22;20:64. doi: 10.1186/s12940-021-00749-4 (PMC8141255; doi:10.1186/s12940-021-00749-4)
Supplement: Supplementary file 1 — Additional file 1. [file 12940_2021_749_MOESM1_ESM.docx]

Supplementary Table 1. Associations between blood cadmium concentrations (per 0.1 μg/L) and reproductive hormones and metabolic markers among non-smokers.

|  | Unadjusted | Adjusted^1^ | Additionally adjusted for rice | Additionally adjusted for grains | Additionally adjusted for green vegetables |
| --- | --- | --- | --- | --- | --- |
|  | %Difference  (95% CI) | %Difference  (95% CI) | %Difference  (95% CI) | %Difference  (95% CI) | %Difference  (95% CI) |
| Reproductive hormones |  |  |  |  |  |
| Testosterone (ng/dL) | 0.8 (-1.1, 2.8) | 2.4 (0.5, 4.4)^*^ | 2.4 (0.5, 4.4)^*^ | 2.5 (0.5, 4.4)^*^ | 2.4 (0.5, 4.4)^*^ |
| Free testosterone (ng/dL) | 0.3 (-2.2, 1.6) | 1.5 (-0.2, 3.3) | 1.5 (-0.2, 3.3) | 1.5 (-0.2, 3.3) | 1.5 (-0.2, 3.3) |
| Free androgen index | -3.0 (-5.7, -0.3)^*^ | -0.4 (-2.9, 2.1) | -0.4 (-2.9, 2.1) | -0.5 (-3.0, 2.0) | -0.5 (-3.0, 2.1) |
| SHBG (nmol/L) | 4.1 (1.4, 6.8)^**^ | 2.8 (0.2, 5.5)^*^ | 2.8 (0.1, 5.5)^*^ | 2.9 (0.3, 5.6)^*^ | 2.9 (0.3, 5.6)^*^ |
| AMH (ng/mL) | 0.8 (-6.2, 8.3) | 8.5 (2.1, 15.2)^**^ | 7.8 (1.5, 14.5)^*^ | 9.4 (2.6, 16.6)^**^ | 8.3 (2.0, 15.1)^**^ |
| Metabolic markers |  |  |  |  |  |
| Insulin (µU/mL) | -1.9 (-4.1, 0.3) | -1.3 (-3.4, 0.7) | -1.3 (-3.3, 0.8) | -1.3 (-3.3, 0.7) | -1.3 (-3.4, 0.7) |
| Glucose (mg/dL) | 0.2 (-0.1, 0.5) | 0.1 (-0.2, 0.4) | 0.1 (-0.2, 0.5) | 0.1 (-0.2, 0.4) | 0.1 (-0.2, 0.5) |
| HOMA-IR | -1.6 (-3.9, 0.7) | -1.2 (-3.4, 1.0) | -1.1 (-3.3, 1.1) | -1.2 (-3.3, 1.0) | -1.2 (-3.3, 1.0) |

^1^ Adjusted for age, BMI, race, and parity.

^*^ P<0.05, ^**^ P<0.01.

AMH, anti Müllerian hormone; BMI, body mass index; CI, confidence interval; HOMA-IR, homeostatic model assessment of insulin resistance; SHBG, sex hormone-binding globulin.
